# Awareness of lung cancer screening among smokers: a Lebanese experience

**DOI:** 10.3389/fonc.2026.1806709

**Published:** 2026-04-15

**Authors:** Karl Rizk, Bachir Atallah, Maissa Safieddine, Joseph Kattan

**Affiliations:** 1 University Medical Center Hotel-Dieu de France, Faculty of Medicine, Saint Joseph University of Beirut, Beirut, Lebanon; 2 Clinical Research Center, Faculty of Medicine Saint Joseph University of Beirut, Beirut, Lebanon; 3 Department of Hematology-Oncology, University Medical Center Hotel-Dieu de France, Faculty of Medicine, Saint-Joseph University of Beirut, Beirut, Lebanon

**Keywords:** awareness, low-dose computed tomography, lung cancer, lung cancer screening, smoking

## Abstract

**Background:**

Lung cancer remains the leading cause of cancer-related mortality worldwide, largely due to late diagnosis, despite strong evidence that low-dose computed tomography (LDCT) reduces mortality and morbidity associated with lung cancer. The effectiveness of screening heavily relies on public awareness and participation. In Lebanon, where tobacco use is prevalent, and no national lung cancer screening program exists, data on LDCT awareness and uptake are limited.

**Objectives:**

This study aims, on one hand, to evaluate awareness and uptake of LDCT screening among Lebanese individuals at risk and, on the other hand, to identify factors associated with knowledge and screening behavior.

**Methods:**

A cross-sectional survey was conducted in Lebanon between December 1, 2024, and December 1, 2025, among current and former Lebanese smokers aged 40 years and older. A structured questionnaire was developed in both online and in-person forms, and collected information on sociodemographic characteristics, smoking history, awareness of LDCT screening, previous screening, and perceived barriers.

**Results:**

242 eligible participants filled the questionnaire. Only 21.1% of participants reported prior knowledge of LDCT screening, and 9.9% had previously undergone screening. The strongest independent predictor of awareness was a higher educational level. LDCT uptake was strongly associated with prior knowledge of the test, male sex, higher socioeconomic status, and active smoking. The most commonly reported barriers included a lack of information and low perceived personal risk, particularly among former smokers.

**Conclusion:**

A significant gap was seen between the proven benefits of LDCT screening and its implementation in Lebanon. These findings highlight the need for education, centered on physicians, targeted public health campaigns, and the formation of a national lung cancer prevention strategy with LDCT to reduce avoidable morbidity and mortality.

## 1 Introduction

Lung cancer remains a great challenge in oncology, with it being the second most frequent cause of cancer and the leading cause of cancer-related mortality globally and in the United States (1). The same pattern is observed in Lebanon, where lung cancer also ranks second in incidence, with breast cancer in the lead (2). In Lebanon, lung cancer represents 9.2% of all cancers diagnosed between 2005 and 2015, with 89% of affected individuals aged 50 and older (89%) (2). Furthermore, Lebanon has one of the highest tobacco burdens in the region, with approximately 37% of adults currently uses tobacco, as per WHO (world health organization) (3). This is particularly relevant, as smoking remains the leading modifiable risk factor, with Lakkis et al. reporting 75.7% of lung cancer cases in males and 66.3% in females are attributable to smoking (4). Therefore, prevention not only requires risk awareness, but also effective secondary prevention strategies in high-risk groups.

Low-dose computed tomography (LDCT) has become the reference screening tool used for lung cancer, in particular high-risk individuals (5). LDCT uses far lower radiation than a normal CT scan with an average of approximately 2mSv according to the National Lung Screening Trial (NLST), and is typically performed without intravenous contrast (6).

Large trials have shown that LDCT screening effectively reduces lung cancer mortality when compared to no screening or screening using chest radiography (7). According to the American Cancer Society, screening is strongly recommended for individuals considered at high risk, with eligibility criteria based on age and cumulative smoking exposure, and typically including current smokers or former smokers who quit within the past 15 years (8). However, guideline quality is not sufficient, the impact of LDCT screening relies also on awareness, acceptability, clinical recommendation, and access. Several studies have documented limited awareness of lung cancer screening, despite high awareness of smoking as a risk factor. Therefore, international surveys have shown that a significant proportion of eligible individuals remain unaware of LDCT screening (9).

Recent data further illustrate this gap. The Lung Health Barometer 2024 reported that general awareness of lung cancer screening remains very low, even though some respondents were willing to undergo screening once informed (10). In that survey, a large proportion of participants were not aware that lung cancer is a leading cause of cancer-related mortality, and results of this survey suggested that a key role in shaping awareness and engagement relies in a patient- provider communication (10). A similar study conducted in Belgium, aiming to assess knowledge and acceptability of lung cancer screening in the general population showed that awareness of LDCT is moderate but there is high acceptability among smoking-related groups, which suggests that screening uptake could improve when programs are clearly explained and implemented (11).

In Lebanon, there is a lack of an organized national screening program and standardized guidelines. National recommendations have recently been proposed by local experts in the form of a joint statement, recommending annual LDCT screening for high-risk individuals aged between 55 and 74 years old with a smoking history of at least 30 pack-years, while also accounting for heavy shisha exposure, a context-specific risk factor locally (12). Harb et al. also underscored the absence of a national lung screening program in Lebanon and reported that undergoing LDCT screening is mainly driven by physician recommendation and patient initiative (13).

Akl et al. showed how important knowing these criteria is to specifically target: only 23.4% of primary care physicians and 14.5% of pulmonary specialist recognized the correct eligibility criteria, while many still used chest X-ray for screening (14). Telvizian et al. studied cancer screening and prevention more broadly in Lebanon across several types of cancer and showed that national screening programs in Lebanon were largely limited by breast cancer (15). This study reported that knowledge about lung cancer screening in heavy smokers was low (15). A more recent study tackled the general lung cancer awareness without assessing LDCT awareness and uptake among smokers and former smokers (16). Although previous Lebanese studies have addressed cancer screening beliefs in the general population and physician knowledge of lung cancer screening, there remains a lack of population-level evidence on awareness and uptake rates of LDCT screening among smokers.

Accordingly, this study aims to assess awareness of lung cancer screening among Lebanese current and former smokers aged 40 years and older, evaluate the proportion of high-risk smokers who have undergone LDCT screening, and identify factors associated with awareness and uptake in order to build strategies to improve screening participation among eligible individuals.

## 2 Methods

### 2.1 Study population and eligibility criteria

The target population consisted of Lebanese nationals aged 40 years or older who are current or former smokers. The lower age limit of 40 years was selected to capture awareness and uptake in a broader at-risk smoking population, including individuals approaching the age range of formal LDCT screening eligibility. A current smoker was defined as any person who currently uses tobacco, regardless of the form (cigarettes, cigars, pipe, or other inhaled products), and a former smoker was defined as an individual who had quit smoking but had a history of regular tobacco use. Given the exploratory nature of the study and the absence of prior Lebanese population- level data on awareness of LDCT screening among smokers, no formal sample size calculation was performed. All eligible participants recruited during the study period were included in the analysis.

### 2.2 Recruitment and data collection

Participants were recruited through two complementary methods: the dissemination of the online questionnaire via social media platforms (Facebook, Instagram, WhatsApp…) and the distribution of the paper version by the study investigators in various public locations, notably the lobby of the University Medical Center Hôtel-Dieu de France, certain public squares frequented by smokers, and high-traffic urban areas. Recruitment happened between December 1, 2024, and December 1, 2025.

### 2.3 Survey and study variables

The questionnaire first collected sociodemographic characteristics, including age, sex, level of education, monthly income, and smoking status. Participants were then asked if they were aware that smoking causes lung cancer. The second section showed a brief explanatory statement LDCT, as follows: *“A low-dose CT scan (or low-dose CT) is an examination recommended annually for people who smoke or have smoked and are at risk of developing lung cancer.”*.

Participants were then asked whether they had heard of or were aware of this lung cancer screening test or not. Those who answered yes were asked if they would be willing to undergo the test.

Participants were subsequently asked if they had ever undergone LDCT for lung cancer screening. If the answer was no, they were asked to indicate the main reason, with multiple-choice options including: lack of knowledge about the test, high cost, fear of the results, concern that the test might be harmful due to radiation exposure, and other reasons.

### 2.4 Outcomes

Two main outcomes were measured: the percentage of high-risk smokers who had already undergone lung cancer screening, and the overall level of awareness of lung cancer screening within the Lebanese smoking population.

### 2.5 Ethical approval

This study was conducted after obtaining approval from the Research Ethics Committee of Saint Joseph University of Beirut (CER-2024-229). Ethical guidelines for research involving human participants were strictly followed, ensuring the protection of privacy and anonymity.

### 2.6 Statistical analysis

All statistical analyses were performed using SPSS software (version 26). A descriptive analysis was first conducted to characterize the study population. Categorical variables (such as sex, education level, and awareness of screening) were presented as frequencies and percentages. Quantitative variables (notably age and duration of smoking) were described using the mean, standard deviation, and their minimum and maximum values. A bivariate analysis was then conducted to identify factors associated, on the one hand, with awareness of lung cancer screening using LDCT and, on the other hand, with actually undergoing this screening. Comparisons were made between participants who had heard about the test and those who had never heard of it, and between those who had already undergone LDCT and those who had never done so. Categorical variables were compared using Pearson’s chi-square test or Fisher’s exact test when required. Quantitative variables were compared using the non-parametric Mann- Whitney test. Variables showing a statistically significant or clinically relevant association in the bivariate analysis were included in a multivariable model. A logistic regression was performed to determine factors independently associated with awareness of LDCT screening. The dependent variable was awareness of screening (yes vs no). The model included education level, income level, and smoking status and its performance was assessed using the omnibus test of model coefficients, Cox and Snell and Nagelkerke pseudo-R² values, the Hosmer–Lemeshow goodness- of-fit test, and the area under the receiver operating characteristic curve (AUC). Results were reported as adjusted odds ratios, along with their 95% confidence intervals and p values and the level of statistical significance was set at p < 0.05.

## 3 Results

### 3.1 General characteristics of participants

**Figure 1:**
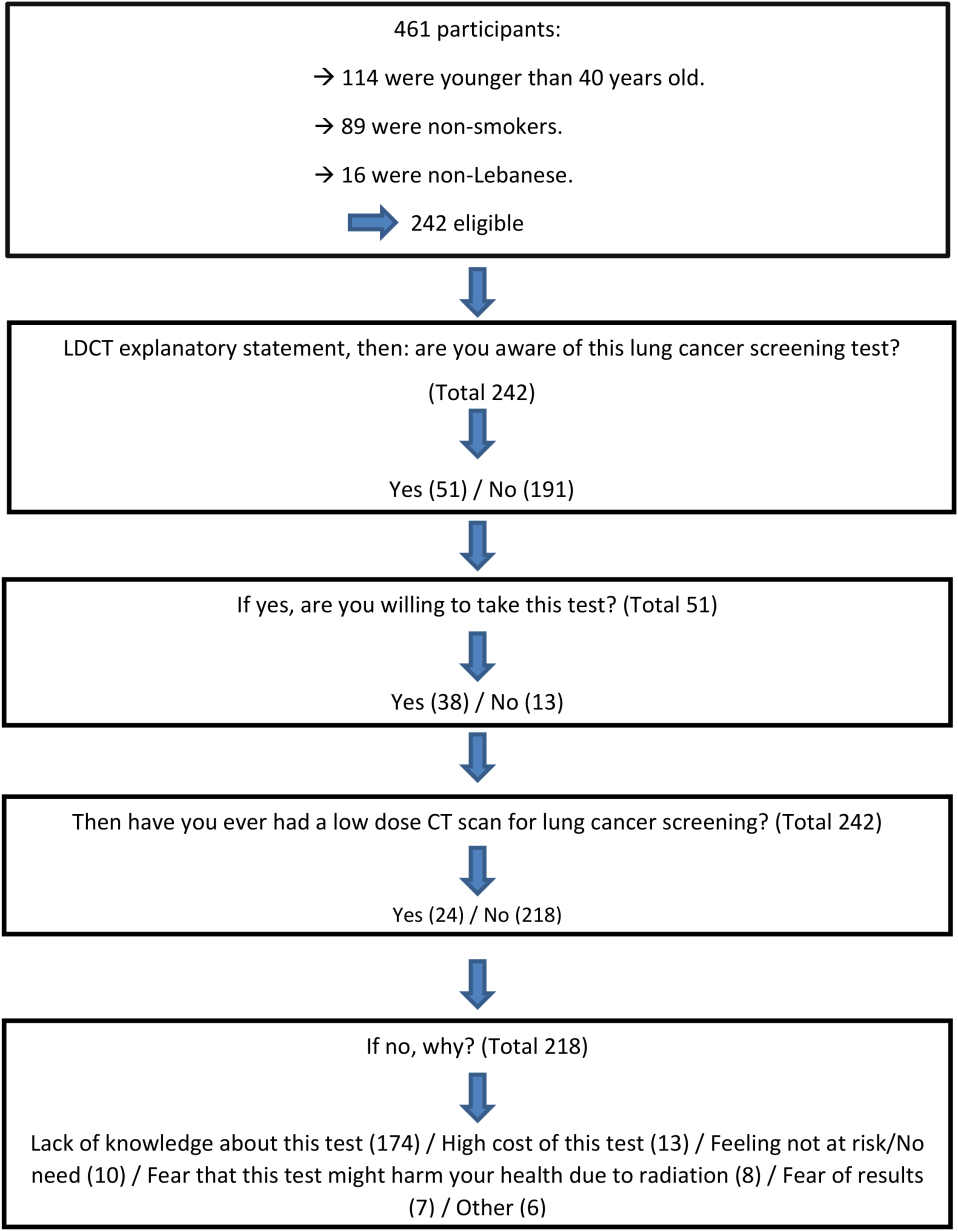
Skip patterns associated with questions pertinent to lung cancer screening.

A total of 461 participants were recruited, of whom 242 met the eligibility criteria. Participant distribution according to their responses is summarized below (cf. **Figure 1**). Among the 242 participants, there was a relatively balanced sex distribution, with 54.1% men and 45.9% women.

The mean age of participants was 59.18 ± 13.25 years. Regarding education, 57% of participants had a low level of education, defined by either uneducated, brevet, secondary school or technical baccalaureate while 43% had a high level of education, defined by bachelor’s degree, master’s degree or doctorate. Income distribution revealed that 41.3% belonged to the low-income category, defined by income < $1000/month compared with 58.7% in the high-income category, defined by revenue > $2500/month. Regarding smoking characteristics, 54.1% of participants were current smokers and 45.9% were former smokers. Mean consumption was 1.14 packs per day, over a mean duration of 29.6 years, resulting in a mean pack-year value of 34.8 (cf. **Table 1**).

**Table 1:** Sociodemographic characteristics, smoking profile, and awareness of lung cancer screening among Lebanese smokers.

Total eligible participants = 242			
Demographics			
Gender		Male	131 (54.1%)
		Female	111 (45.9%)
Age		Mean (SD)	59.18 (13.25)
		Min - Max	40 – 91
		40-49	67 (27.7%)
		50-59	66 (27.3%)
		60-69	50 (20.7%)
		>69	59 (24.4%)
Education	Lower (57.0%)	Uneducated	20 (8.3%)
		Secondary School	54 (22.3%)
		Brevet	22 (9.1%)
		Technical Baccalaureate	42 (17.4%)
	Higher (43.0%)	Bachelor’s Degree	47 (19.4%)
		Master’s Degree	52 (21.5%)
		Doctorate	5 (2.1%)
Income	Lower (41.3%)	< 500$	53 (21.9%)
		500 - 1000$	47 (19.4%)
	Higher (58.7%)	1000 - 1500$	37 (15.3%)
		1500 - 2000$	32 (13.2%)
		2000 - 2500$	30 (12.4%)
		> 2500$	43 (17.8%)
Smoking related characteristics			
Smoking related characteristics		Former smoker	111 (45.9%)
		Active smoker	131 (54.1%)
Pack years		Mean (SD)	34.78 (22.69)
Total eligible participants = 242			
		Min - Max	3 - 130
Lung cancer screening awareness			
Are you aware that smoking significantly increases the risk of developing lung cancer?		No	6 (2.5%)
		Yes	236 (97.5%)
Have you heard about this lung cancer screening test?		No	191 (78.9%)
		Yes	51 (21.1%)
If yes, are you willing to take this test?		No	13 (25.5%)
		Yes	38 (74.5%)
Have you ever had a low dose CT scan for lung cancer screening?		No	218 (90.1%)
		Yes	24 (9.9%)
If not, why?		Lack of knowledge about this test	174 (79.8%)
		Fear that this test might harm your health due to radiation	8 (3.7%)
		High cost of this test	13 (6.0%)
		Fear of the results	7 (3.2%)
		Did not feel at risk/No need	10 (4.6%)
		Others	6 (2.8%)

LDCT, low-dose computed tomography; SD, Standard deviation. Data are presented as n (%) unless otherwise indicated.

### 3.2 Awareness of risk and screening

Almost all participants (97.5%) reported being aware that smoking significantly increases the risk of lung cancer. However, only 21.1% of respondents had heard of LDCT, while 78.9% had never heard of it. Among those who were aware of the existence of the test, 74.5% stated that they were willing to undergo it (cf. **Table 1**).

### 3.3 Factors associated with awareness of screening

Bivariate analysis revealed that awareness of LDCT was more frequent among participant with higher education level than those with a lower level of education (68.6% vs 31.4%, p < 0.001). Information about LDCT screening was also more frequent with higher-income than those with lower-income (74.5% vs 25.5%, p = 0.010). Smoking status was not significantly associated with awareness of LDCT screening, although a higher proportion of current smokers had heard of screening compared with former smokers (64.7% vs 35.3%, p = 0.088). In contrast, neither age nor sex was associated with awareness of the test. Age and sex were not significantly associated with awareness (cf. **Table 2**).

**Table 2:** Factors associated with awareness of lung cancer screening by LDCT among Lebanese smokers.

Variables		Total (N = 242)	Never heard about Low dose CT scan (N = 191)	Heard about this lung cancer screening test (N = 51)	P value
Gender	Male	131 (54.1%)	104 (54.5%)	27 (52.9%)	0.848
	Female	111 (45.9%)	87 (45.5%)	24 (47.1%)	
Age (in years)	Mean (SD)	59.18 (13.25)	59.72 (13.46)	57.18 (12.35)	0.224
	Min - Max	40 - 91	40 - 91	40 - 88	
Education	Lower	138 (57.0%)	122 (63.9%)	16 (31.4%)	**<0.001**
	Higher	104 (43.0%)	69 (36.1%)	35 (68.6%)	
Income	Lower	100 (41.3%)	87 (45.5%)	13 (25.5%)	**0.010**
	Higher	142 (58.7%)	104 (54.5%)	38 (74.5%)	
Active smoker	Former smoker	111 (45.9%)	93 (48.7%)	18 (35.3%)	0.088
	Active smoker	131 (54.1%)	98 (51.3%)	33 (64.7%)	
Are you aware that smoking significantly increases the risk of developing lung cancer?	No	6 (2.5%)	6 (3.1%)	0 (0.0%)	0.348
	Yes	236 (97.5%)	185 (96.9%)	51 (100.0%)	

LDCT, low-dose computed tomography; SD, Standard deviation. P values were calculated using Pearson’s chi-square test or Fisher’s exact test for categorical variables and the Mann–Whitney U test for continuous variables, as appropriate.

In multivariable logistic regression, higher education remained independently associated with awareness (OR = 3.64). Current smoking was also independently associated with awareness (OR= 1.97). In contrast, income was not statistically significant (cf. **Table 3**).

**Table 3:** Multivariable analysis: factors independently associated with awareness of lung cancer screening by LDCT.

	OR [95% CI]	P value
Education	3.638 [1.695 - 7.805]	**0.001**
Income	1.301 [0.580 - 2.920]	0.524
Smoking	1.967 [1.006 - 3.846]	**0.048**
a. Dependent variable: Have you heard about this lung cancer screening test?		
b. Variable(s) entered in the model: - Education (1: Lower/2: Higher) - Smoking (0: Former smoker/1: Active smoker)		

OR, odds ratio; CI, confidence interval; LDCT, low-dose computed tomography.

Multivariable logistic regression was performed with awareness of LDCT screening as the dependent variable. Variables entered into the model were education level, income level, and smoking status. Model performance: omnibus χ² = 21.879, p < 0.001; Cox and Snell R² = 0.086; Nagelkerke R² = 0.134; Hosmer–Lemeshow p = 0.968; AUC = 0.702 (95% CI: 0.620–0.785).

The multivariable logistic regression model was statistically significant overall (omnibus χ² = 21.879, df = 3, p < 0.001). The model showed modest explanatory power, with a Cox and Snell R² of 0.086 and a Nagelkerke R² of 0.134. Calibration was satisfactory, with a non-significant Hosmer–Lemeshow test (χ² = 1.356, df = 6, p = 0.968). Discriminatory ability was acceptable, with an area under the receiver operating characteristic curve of 0.702 (95% CI: 0.620–0.785, p < 0.001).

### 3.4 Factors associated with undergoing screening

Only 9.9% of participants having undergone LDCT screening. In bivariate analysis, male sex participants were significantly associated with having undergone the test (75.0% vs 51.8%, p = 0.031), as were individuals with a high level of education (83.3% vs 16.7%, p = 0.009), same with participants with a high income (70.8% vs 29.2%, p = 0.004), current smokers (83.3% vs 16.7%, p= 0.002), and prior awareness of the (79.2% vs 14.7%, p < 0.001) (cf. **Table 4**). No multivariable model was performed for this outcome because only 24 participants reported prior screening, which was considered insufficient for a stable adjusted analysis.

**Table 4:** Factors associated with undergoing a LDCT for lung cancer screening among Lebanese smokers.

Variables		Total (N = 242)	Never had a low dose CT scan (N = 218)	Had a low dose CT scan (N = 24)	P value
Gender	Male	131 (54.1%)	113 (51.8%)	18 (75.0%)	**0.031**
	Female	111 (45.9%)	105 (48.2%)	6 (25.0%)	
Age (in years)	Mean (SD)	59.18 (13.25)	59.72 (13.46)	57.18 (12.35)	0.670
	Min - Max	40 - 91	40 - 91	41 - 81	
Education	Lower	138 (57.0%)	131 (60.1%)	7 (29.2%)	**0.004**
	Higher	104 (43.0%)	87 (39.9%)	17 (70.8%)	
Income	Lower	100 (41.3%)	96 (44.0%)	4 (16.7%)	**0.009**
	Higher	142 (58.7%)	122 (56.0%)	20 (83.3%)	
Active smoker	Former smoker	111 (45.9%)	107 (49.1%)	4 (16.7%)	**0.002**
	Active smoker	131 (54.1%)	111 (50.9%)	20 (83.3%)	
Are you aware that smoking significantly increases the risk of developing lung cancer?	No	6 (2.5%)	6 (2.8%)	0 (0.0%)	1.000
	Yes	236 (97.5%)	212 (97.2%)	24 (100.0%)	
Have you heard about this lung cancer screening test?	No	191 (78.9%)	186 (85.3%)	5 (20.8%)	**<0.001**
	Yes	51 (21.1%)	32 (14.7%)	19 (79.2%)	

LDCT, low-dose computed tomography; SD, Standard deviation.

P values were calculated using Pearson’s chi-square test or Fisher’s exact test for categorical variables and the Mann–Whitney U test for continuous variables, as appropriate.

## 4 Discussion

Lebanon faces a particularly heavy tobacco burden, with significantly high levels of both cigarette and especially water-pipe smoking in the adult population (17), contributing to the highest incidence of lung cancer for females and the second highest for males in the MENA (Middle-East and North Africa) region (18). These findings highlight the importance of strengthening lung cancer screening efforts to reduce the impact of this substantial burden.

In this study, awareness of LDCT screening was low among Lebanese current and former smokers aged 40 years and older, despite the proven benefit of this modality in reducing lung cancer mortality (19–21). Although nearly all participants recognized the strong association between smoking and lung cancer, only a small proportion had heard of LDCT, and an even smaller proportion had ever undergone screening. A key finding was that risk perception and knowledge of secondary prevention were markedly disconnected. While 97.5% of participants acknowledged smoking as a major cause of lung cancer, only 21.1% were aware of LDCT screening. This descriptive contrast highlights an important gap between general awareness of smoking-related risk and knowledge of available screening options. However, our study was not designed to formally test the association between these two constructs.

Similar observations have been reported internationally. In the United States, Sharma et al. found that only about half of smokers and former smokers were aware of LDCT screening despite widespread knowledge of smoking-related risk (9). Likewise, the Community Awareness of Lung Cancer Screening study showed that poor knowledge of LDCT remains a major barrier to screening participation even in populations with high general awareness of lung cancer (22). European reviews have also highlighted limited dissemination of information in high-risk groups, constraining the population impact of screening programs (23).

Of note, apparent inconsistencies in self-reported awareness and screening in our study: because awareness and prior screening were self-reported, some participants may have confused LDCT performed for screening with a standard chest CT performed for diagnostic purposes. This potential misclassification may have overestimated both awareness and uptake. This possibility may have been more pronounced in self-administered survey formats, where real-time clarification was not available. These findings may underscore substantial gaps in communication and education regarding lung cancer screening, both within the healthcare system and among the general population.

Despite low awareness, acceptability of LDCT was high once participants were informed, since nearly three-quarters (74.5%) of those who knew about the test expressed willingness to undergo screening. This shows that poor uptake is mainly limited by the lack of information and medical recommendation rather than patient reluctance.

International evidence strongly supports this interpretation. Carter-Harris et al. showed that physician recommendation is one of the strongest predictors of LDCT uptake, independent of perceived risk (24), and Sedani et al. reported that lack of clinician to patient discussion is a major barrier even in settings with national guidelines (25). A systematic review by Field et al. similarly demonstrated that insufficient engagement of healthcare professionals substantially limits the effectiveness of LDCT screening programs in Europe (26).

Education level emerged as the strongest independent determinant of awareness, with individuals with higher education being significantly more likely to know about LDCT screening. However, because of the cross-sectional design, the temporal direction of this relationship cannot be determined. It is possible that education serves as a proxy for broader factors such as health literacy, healthcare access, information exposure, or engagement with preventive care. Although the logistic regression model was statistically significant and showed acceptable discrimination, its pseudo-R² values were modest, suggesting that additional unmeasured factors may contribute to awareness of LDCT screening in this population. This finding highlights important inequalities in health literacy and access to preventive information. Castro et al. showed that lower education and income are associated with poorer understanding of screening eligibility and lower participation in LDCT programs (27).

Further analyses of social determinants of health confirm that socioeconomic disadvantage, lack of insurance, and financial insecurity substantially reduce engagement in cancer screening (28). These patterns are also evident in our cohort, where current smokers were more informed than former smokers, which is a concerning finding given that former smokers constitute a major target group in screening guidelines (8).

Regarding actual screening uptake, male sex, higher socioeconomic status, and current smoking were associated with a greater likelihood of undergoing LDCT. These results align with international data showing that both physician recommendation and financial access are critical drivers of screening behavior. Studies have demonstrated that patients who receive a clear recommendation from their physician are far more likely to undergo LDCT than those who do not (29), while cost concerns and lack of insurance remain major obstacles even among informed individuals (30). A systematic review further confirmed that access to healthcare services and interactions with healthcare professionals are key to actually change screening behavior and turn knowledge into action (31).

Participants in our study who had not undergone screening most frequently cited lack of knowledge (79.8%), perceived cost (6%), low perceived personal risk (4.6%), fear of radiation (3.7%), and fear of results (3.2%). These barriers are largely modifiable. Radiation concerns persist despite strong evidence that LDCT delivers a substantially lower dose than standard CT and offers a favorable benefit-risk ratio in high-risk populations (19–21). Financial barriers and uncertainty about reimbursement have also been repeatedly identified as limiting screening uptake (29, 30), while anxiety about a possible cancer diagnosis can discourage participation (22).

In Lebanon, the absence of a structured national screening program contributes substantially to the low awareness and uptake observed with LDCT screening, where it remains dependent on individual initiative or occasional physician recommendation. A recent survey of Lebanese physicians showed that although many recognize the effectiveness of LDCT, a significant proportion do not correctly identify eligibility criteria and continue to rely on non-recommended methods such as chest radiography (14).

In contrast, several European countries have started to develop pilot and national screening programs based on interdisciplinary collaboration and targeted strategies to reach high-risk populations (32). In the United States, LDCT screening is supported by public health recommendations and partial insurance coverage. However, participation rates remain relatively low (33, 34). These observations emphasize the importance of adopting a coordinated and comprehensive approach in Lebanon, combining public awareness campaigns, improved training of healthcare professionals, and policies aimed at reducing financial and organizational barriers to lung cancer screening.

Next to the small number of eligible participants, several limitations should be considered when interpreting the findings. The cross-sectional design does not allow for causal relationships to be established. Selection bias may have occurred, as individuals who chose to participate could have been more concerned about health-related issues than the general smoking population.

Moreover, the reliance on self-reported data introduces the possibility of recall bias and social desirability bias. Confusion between standard chest CT and LDCT may also have resulted in an overestimation of both awareness and screening uptake with the possibility of outcome misclassification. In addition to education and income, awareness and uptake of LDCT screening may be influenced by unmeasured factors such as physician recommendation, access to healthcare services, and insurance coverage, area of residence, proximity to healthcare facilities, transportation barriers, prior medical history, family history of cancer, health literacy, and exposure to health information through media or social networks. Because these variables were not collected, the observed associations should be interpreted with caution.

Willingness to undergo LDCT screening was only assessed among participants already aware of LDCT screening. Therefore, the study could not evaluate the hypothetical acceptability among previously unaware individuals after receiving the standardized explanation.

The generalizability of our findings is limited. Participants were recruited through a combination of online dissemination and in-person sampling in selected public and hospital-affiliated settings, which may not accurately reflect the broader Lebanese population of smokers and former smokers. This sample may over represent individuals with better healthcare contact, urban residence, or higher digital access.

We acknowledge that the inclusion of participants aged between 40 and 49 years extends below the age range currently recommended for routine LDCT screening and may therefore limit direct comparability with screening-eligible populations. The age structure of the sample may also have been influenced by the recruitment approach. Online dissemination may have reduced participation among older adults with lower digital literacy or limited use of social media, potentially affecting representativeness across age groups. To reduce this bias, paper questionnaires were also distributed in public and hospital-based settings. However, age-related participation bias cannot be excluded. Another limitation is that no upper age limit was applied. As current screening recommendations generally apply to individuals within a defined upper age range, inclusion of participants older than this range may limit direct interpretation of screening uptake relative to eligible populations.

Despite these limitations, this study offers valuable and novel insights into a topic that has received little attention in Lebanon and the Middle East region. It provides a strong foundation for future research, particularly longitudinal studies that incorporate medical interviews and objective clinical data.

## 5 Conclusion

Although the vast majority of Lebanese participants were aware of the strong link between smoking and lung cancer, awareness of LDCT screening remains very limited, with education level emerging as the strongest independent factor associated with knowledge of the test. These findings underscore the need for broader and more coordinated efforts to better understand and address the major gaps in both awareness and uptake of lung cancer screening in Lebanon.

Large, multicenter studies conducted across different regions of the country would help improve the representativeness of the data and confirm the trends observed in this study. In parallel, the development of strategies adapted to the Lebanese context, especially considering economic challenges, the absence of a national screening program, and existing inequalities in access to healthcare is essential to enhance awareness and facilitate access to lung cancer screening.

## Data Availability

The original contributions presented in the study are included in the article/**Supplementary Material**. Further inquiries can be directed to the corresponding author.
